# Mutations of Conserved Residues in the Major Homology Region Arrest Assembling HIV-1 Gag as a Membrane-Targeted Intermediate Containing Genomic RNA and Cellular Proteins

**DOI:** 10.1128/JVI.02698-15

**Published:** 2016-01-28

**Authors:** Motoko Tanaka, Bridget A. Robinson, Kasana Chutiraka, Clair D. Geary, Jonathan C. Reed, Jaisri R. Lingappa

**Affiliations:** a Department of Global Health, University of Washington, Seattle, Washington, USA; b Departments of Medicine and Microbiology, University of Washington, Seattle, Washington, USA

## Abstract

The major homology region (MHR) is a highly conserved motif that is found within the Gag protein of all orthoretroviruses and some retrotransposons. While it is widely accepted that the MHR is critical for assembly of HIV-1 and other retroviruses, how the MHR functions and why it is so highly conserved are not understood. Moreover, consensus is lacking on when HIV-1 MHR residues function during assembly. Here, we first addressed previous conflicting reports by confirming that MHR deletion, like conserved MHR residue substitution, leads to a dramatic reduction in particle production in human and nonhuman primate cells expressing HIV-1 proviruses. Next, we used biochemical analyses and immunoelectron microscopy to demonstrate that conserved residues in the MHR are required after assembling Gag has associated with genomic RNA, recruited critical host factors involved in assembly, and targeted to the plasma membrane. The exact point of inhibition at the plasma membrane differed depending on the specific mutation, with one MHR mutant arrested as a membrane-associated intermediate that is stable upon high-salt treatment and other MHR mutants arrested as labile, membrane-associated intermediates. Finally, we observed the same assembly-defective phenotypes when the MHR deletion or conserved MHR residue substitutions were engineered into Gag from a subtype B, lab-adapted provirus or Gag from a subtype C primary isolate that was codon optimized. Together, our data support a model in which MHR residues act just after membrane targeting, with some MHR residues promoting stability and another promoting multimerization of the membrane-targeted assembling Gag oligomer.

**IMPORTANCE** The retroviral Gag protein exhibits extensive amino acid sequence variation overall; however, one region of Gag, termed the major homology region, is conserved among all retroviruses and even some yeast retrotransposons, although the reason for this conservation remains poorly understood. Highly conserved residues in the major homology region are required for assembly of retroviruses; however, when these residues are required during assembly is not clear. Here, we used biochemical and electron microscopic analyses to demonstrate that these conserved residues function after assembling HIV-1 Gag has associated with genomic RNA, recruited critical host factors involved in assembly, and targeted to the plasma membrane but before Gag has completed the assembly process. By revealing precisely when conserved residues in the major homology region are required during assembly, these studies resolve existing controversies and set the stage for future experiments aimed at a more complete understanding of how the major homology region functions.

## INTRODUCTION

In HIV-1-infected cells, viral Gag proteins assemble into a spherical immature lattice (also called an immature capsid) before undergoing budding and maturation. This process leads to release of fully infectious mature virus particles, each of which contains ∼3,000 Gag proteins. During assembly, HIV-1 Gag is a 55-kDa polyprotein that contains four domains (matrix [MA], capsid [CA], nucleocapsid [NC], and p6) and two spacer peptides; subsequently, during maturation, Gag is cleaved into separate proteins by the HIV-1 protease. Each domain of Gag plays an important role in immature virus production, with the first three domains being involved in assembly of the immature lattice, and the p6 domain being required for proper budding and release. MA is critical for membrane targeting of assembling Gag, CA is involved in Gag multimerization, and NC is involved in both encapsidating viral genomic RNA (gRNA) and initiating Gag-Gag contacts via nonspecific RNA interactions (reviewed in reference 1).

**FIG 1:**
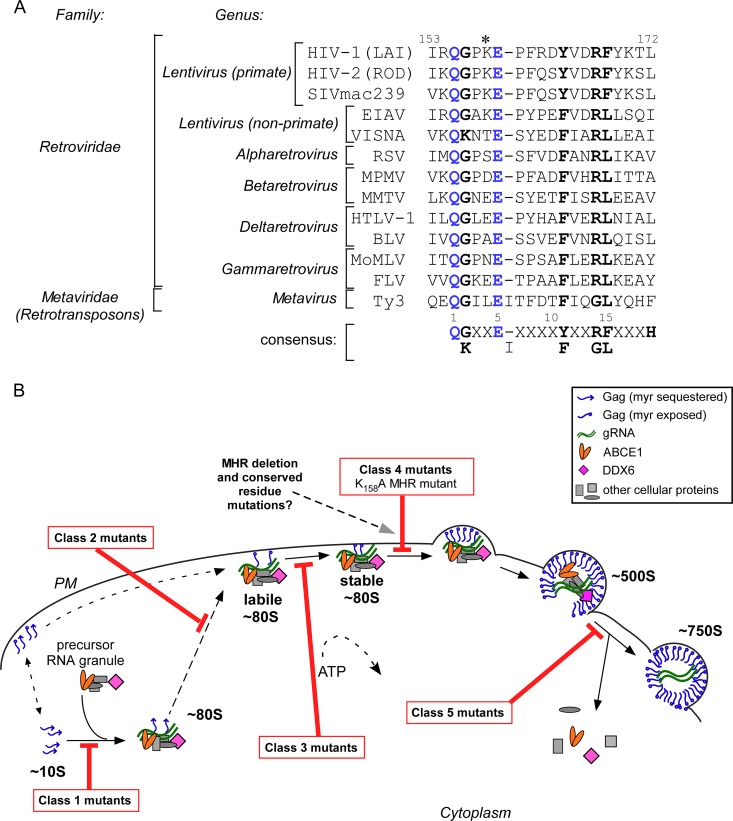
MHR alignment and location of known blocks in the HIV-1 assembly pathway. (A) Alignment of the amino acid sequences of the MHRs of retroviruses and the Ty3 yeast retrotransposon, with retrovirus or retrotransposon family and genus indicated. Amino acid numbering of the MHR within the HIV-1 capsid protein is shown above. The 19-residue consensus sequence for the MHR, based on the 13 sequences shown, is indicated with motif numbers above. Note that H represents hydrophobic residues. Invariant amino acids are shown in blue, highly conserved amino acids are in bold black, and the previously studied K158A mutation (7, 31) is indicated with an asterisk. SIV, simian immunodeficiency virus; EIAV, equine infectious anemia virus; VISNA, visna virus; RSV, Rous sarcoma virus; MPMV, Mason-Pfizer monkey virus; MMTV, mouse mammary tumor virus; HTLV-1, human T-cell lymphotropic virus type 1; BLV, bovine leukemia virus; MoMLV, Moloney murine leukemia virus; FLV, feline leukemia virus. (B) Diagram showing the hypothesis tested here, in the context of the HIV-1 assembly pathway. Newly synthesized Gag forms the ∼10S intermediate, which is recruited into the host precursor complex to form the ∼80S cytosolic assembly intermediate. The ∼80S intermediate likely traffics to the PM, where it forms the ∼150S and ∼500S assembly intermediates. The ∼80S, ∼150S, and ∼500S assembly intermediates are associated with the cellular facilitators of assembly ABCE1 and DDX6, while ∼10S Gag is not. Upon formation of the completely assembled ∼750S completed immature capsid, cellular proteins from the host precursor complex dissociate. Note that ∼10S Gag is also found at the PM and may be recruited into assembling ∼80S intermediates at the PM. Dashed lines indicate steps whose location or progression is hypothetical. For example, the pathway is ATP dependent, but exactly where ATP is required remains to be determined. Known assembly-defective Gag mutants are arrested at five different steps in the assembly pathway (classes 1 to 5, indicated by red bars showing points of blockade), as described in the text and reviewed in reference 1. Here, we hypothesized that MHR deletion and conserved residue mutations are class 4 mutations, like another MHR mutation tested previously (K158A).

While a high-resolution structure has not yet been obtained for full-length Gag, such structures have been obtained for MA, the N-terminal subdomain of CA (CA-NTD), and the C-terminal subdomain of CA (CA-CTD) of HIV-1 and other retroviruses. The structure of each of these domains is well conserved across retroviruses despite poor overall conservation of their respective primary amino acid sequences (reviewed in reference 2). Notably, amid the limited amino acid sequence conservation of Gag, two Gag regions stand out for being highly conserved—NC and the major homology region (MHR). While the function of the NC region is relatively well understood (reviewed in references 1 and 3), the exact function of the highly conserved MHR remains unclear, despite intensive study, as noted by others (4). The MHR is an 18- to 19-amino-acid motif in CA that is present in all orthoretroviruses and in the yeast retrotransposon Ty3, which is distantly related to retroviruses (Fig. 1A). Indeed, the MHR consensus (shown in Fig. 1A) is so characteristic of a Gag sequence that its presence allows identification of new endogenous retrovirus sequences in host genomes (e.g., see reference 5). Moreover, the only regions of HIV-1 Gag that are absolutely required for assembly (i.e., they cannot be deleted or replaced with heterologous domains) are the CA-CTD subdomain, which contains the MHR, and the adjacent sp1 spacer (6), further emphasizing the importance of the MHR for Gag assembly. Structural analyses reveal that MHR residues form a critical intrahexameric interface in the completed immature capsid (7–9), as described in detail in Discussion, indicating that MHR residues are important for capsid structure. However, the structural importance of the MHR residues does not, on its own, explain the high degree of MHR conservation, since other regions of Gag (such as CA helix 9) also form key interfaces in the fully assembled immature capsid (10) but do not display the same degree of amino acid conservation. Thus, despite many years of study, a compelling explanation is lacking for why the MHR motif displays such a significant degree of conservation across such a wide evolutionary spectrum.

There is general agreement that residues in the MHR are important for immature lattice assembly, since MHR mutations lead to failed or aberrant assembly for HIV-1 (11–14), Rous sarcoma virus (RSV), Mason-Pfizer monkey virus, bovine leukemia virus, and the Ty3 yeast retrotransposon (15–18). Consistent with these findings, yeast two-hybrid studies suggest that the HIV-1 MHR is important for multimerization (19). Additionally, some MHR mutations do not inhibit virus or capsid production but cause defects at other stages in the life cycle: for example, reduced infectivity and maturation of RSV (15, 20, 21), reduced infectivity of HIV-1 (22), and reduced retrotransposition of Ty3 (16). Notably, defects subsequent to assembly could result from entirely distinct functions of the MHR or from abnormalities in capsid assembly that manifest only later, such as after entry. Regarding other possible functions of the MHR, it should be noted that MHR residues cross-link to the maturation inhibitor bevirimat (23) and also govern resistance to and dependence on a different maturation inhibitor, PF-46396 (24). In addition, the MHR of the cleaved CA protein of murine leukemia virus (MLV) binds to an enzyme that mediates sumoylation of CA required for postentry events (25), although a role for the MHR of other retroviruses in sumoylation has not been reported.

In the current study, we focused on the role of the MHR in assembly. Despite a consensus that conserved residues in the MHR are critical for assembly of the immature capsid of HIV-1 and other retroviruses, there is surprisingly little agreement on when these residues act during assembly. Specifically, one study reported that MHR mutations cause defects in membrane targeting of Gag (11), while another reported that point mutations of a conserved MHR residue inhibit assembly after membrane targeting of Gag (7). Additionally, while there is agreement regarding the deleterious effect of conserved MHR residue point mutations on virus-like particle (VLP) production (7, 11, 13), conflicting results have been reported regarding the effect of MHR deletion, with studies finding either a dramatic (11) or modest 50% (26) reduction in VLP production. Thus, despite the importance of this conserved motif, there is ambiguity about the exact phenotype of the HIV-1 MHR deletion and the stage at which various MHR point mutations inhibit assembly.

Here, we sought to resolve these controversies and gain new insights into MHR function by identifying the exact step in HIV-1 assembly at which highly conserved MHR residues are required. In the case of HIV-1, the recent delineation of a temporospatial assembly pathway allows the assembly defect exhibited by MHR mutants to be defined in detail. In cells, newly synthesized HIV-1 Gag transits through a series of assembly intermediates before forming the fully assembled immature capsid, which has a sedimentation value of ∼750S (Fig. 1B). Evidence that these Gag-containing complexes, which are named by their sedimentation values (∼10S, ∼80S, ∼150S, and ∼500S), are assembly intermediates came initially from pulse-chase experiments (27, 28). Additional support for the order of these sequential intermediates was provided by mutational analyses showing that each known assembly-defective Gag mutant is arrested at a specific point along this pathway, with accumulation of Gag in the form of assembly intermediates that precede the block (27, 29–31). In addition to containing Gag, the ∼80S, ∼150S, and ∼500S assembly intermediates (termed the high-molecular-weight intermediates) contain the HIV-1 genome (31) as well as cellular proteins (27, 29, 30, 32, 33). Among the cellular proteins present in these complexes are two proteins known to facilitate HIV-1 capsid assembly, the ATP-binding cassette protein E1 (ABCE1) and the DEAD box RNA helicase DDX6, also known as RCK/p54 (32, 33). These cellular proteins are recruited into assembly intermediates when ∼10S Gag coopts a cellular RNA granule that contains ABCE1, DDX6, argonaute-2 (AGO2), and other cellular proteins (32). This results in formation of the first high-molecular-weight assembly intermediate, the ∼80S complex. The ∼80S intermediate contains Gag as well as gRNA and is found both in the cytoplasm and at the plasma membrane (PM) (31); thus, it fits the description of the complex observed to anchor assembling Gag at the PM in live imaging studies (34) (Fig. 1B). Further Gag multimerization at the PM leads to the formation of the ∼150S and ∼500S intermediates, which are found exclusively at the PM (31).

When the HIV-1 provirus is expressed in cells, well-studied assembly-defective HIV-1 Gag mutants fall into five classes based on where they inhibit the HIV-1 assembly pathway described above (reviewed in reference 1) (Fig. 1B). Two types of Gag mutants are arrested at the first step in the assembly pathway (class 1), forming only the ∼10S intermediate. These include Gag proteins that are truncated just after CA as well as full-length Gag proteins containing nine or more NC basic residue substitutions (35). Mutations of residues critical for Gag myristoylation or myristate exposure (e.g., G2A, V7R, or L8A in MA) arrest Gag at the second step in the assembly pathway (class 2), with no progression past the cytosolic ∼80S assembly intermediate (29, 31). Point mutations in CA helix 9 (e.g., WM184/185AA), which forms the CA dimer interface (30, 31), arrest Gag at the next step in the assembly pathway, as a labile (salt-sensitive), membrane-associated ∼80S intermediate (class 3). In contrast, point mutations of residues located in three different regions of CA that form the “base” of the CA-CTD crystal structure (K158A, P224A, and D197A in CA-CTD) arrest Gag as a stable (salt-insensitive), membrane-bound ∼80S assembly intermediate (class 4) (31). Finally, point mutations in helices 4 to 6 in CA-NTD cause arrest of Gag at the last step in the assembly pathway, the ∼500S step (class 5) (31).

One of the mutations that we had analyzed previously (K158A) is located in the HIV-1 MHR. K158 is not a highly conserved MHR residue since it is present in many but not all lentiviruses and is not found in prototype alpha-, beta-, delta-, and gammaretroviruses (Fig. 1A). K158 was studied previously because alanine-scanning mutagenesis had revealed it to be critical for immature HIV-1 particle production (7). Our finding that K158A is a class 4 mutation, arresting Gag after formation of a stable, membrane-targeted intermediate (31) (Fig. 1B), is consistent with electron microscopy (EM) images generated by one group (7) but at odds with a report suggesting that K158A and mutations of conserved MHR residues inhibit membrane targeting of Gag (11).

Thus, as described above, it remains unclear whether MHR deletion significantly inhibits virus assembly, whether mutation of conserved MHR residues arrests assembling HIV-1 Gag before or after membrane targeting, and whether point mutations of highly conserved MHR residues and the less conserved K158A residue yield the same class 4 assembly phenotype. To resolve these questions and to pinpoint the exact step(s) in assembly at which conserved MHR residues are required, we analyzed assembly defects exhibited by Gag constructs containing an MHR deletion or one or more point mutations of highly conserved MHR residues. Our biochemical and ultrastructural studies demonstrate that the MHR deletion and all the MHR point mutations that we have tested to date arrest assembling Gag after membrane targeting but before early budding forms are generated, in both 293T and COS-1 cells. Interestingly, the exact point of arrest at the PM differed depending on the specific MHR mutation, with MHR deletion and point mutations of highly conserved residues resulting in class 3 arrest, while the previously described K158A mutation resulted in class 4 arrest. All of the arrested MHR mutants that we examined were associated with genomic RNA as well as the cellular proteins ABCE1 and DDX6. The same phenotype was observed upon mutation of one, two, or four of the invariant and/or highly conserved residues in the MHR, indicating that the defects are not additive. Additionally, introducing the MHR mutations into a codon-optimized, subtype C Gag background resulted in the same assembly defects. Together, these studies resolve existing controversies, define the exact assembly defect caused by deletion and conserved residue mutations in the HIV-1 MHR, and provide insights into how the MHR functions during assembly.

## MATERIALS AND METHODS

### Cells and plasmids.

Constructs utilized in this study were generated via site-directed mutagenesis in one of two plasmids: an HIV-1 provirus, LAI strain, with a deletion in *env* and an inactive protease described previously (31) (NCBI nucleotide accession number K02013.1), or a codon-optimized, type C, primary isolate Gag obtained from the NIH AIDS Reagent Program (catalog no. 8675; p96ZM651gag-opt from Yingying Li, Feng Gao, and Beatrice H. Hahn [36]; NCBI nucleotide accession number AY181195). Sequences of oligonucleotides used to generate each mutation are available upon request. COS-1 and 293T cells were obtained from ATCC and maintained in Dulbecco modified Eagle medium (DMEM) (Life Technologies) with 10% fetal bovine serum (FBS). Sequence identity and homology for HIV-1 LAI (subtype B, lab adapted) with subtype C primary isolate Gag were determined using Clustal W, Gonnet similarity matrix.

### Transfection, immunoprecipitations, and Western blot analyses.

COS-1 or 293T cells were transfected with 1 to 5 μg DNA using polyethylenimine (Polysciences, Warrington, PA). Cell lysates were harvested in the presence of freshly prepared protease inhibitor cocktail (Sigma, St. Louis, MO) and RNaseOUT (Invitrogen), in 1× Triton-X buffer (20 mM HEPES, pH 7.9, 10 mM NaCl, 10 mM EDTA, pH 8.0, 0.35% Triton X-100) followed by immunoprecipitation with antibody to ABCE1 (33) or antibody to DDX6 (Bethyl, Montgomery, TX), using protein G-coupled Dynabeads (Life Technologies), as described previously (32). Immunoprecipitation eluates were analyzed by SDS-PAGE, followed by Western blot (WB) analysis using HIV immunoglobulin (NIH AIDS Reagents catalog no. 3957, from NABI and NHLBI) with a horseradish peroxidase (HRP)-tagged anti-human IgG secondary antibody (Bethyl Laboratories, Montgomery, TX) or an antibody to HIV-1 MA (Capricorn, Portland, ME), and with an HRP-tagged anti-mouse IgG1 secondary antibody (Santa Cruz, Dallas, TX). Immunoprecipitation eluates were routinely reprobed with antibody to ABCE1, followed by HRP-tagged protein A (Thermo Fisher Scientific, Rockford, IL) to confirm ABCE1 immunoprecipitation (data not shown). WB signals from immunoprecipitation eluates were detected using Pierce ECL substrate (Thermo Fisher Scientific) with Carestream Kodak Biomax Light film. For detection of Gag in total cell lysates, velocity sedimentation fractions, and membrane flotation fractions, WB analyses were performed as described above or using antibodies conjugated to infrared dyes (Li-Cor, Lincoln, NE). Quantification of Gag bands on film was performed using ImageJ software.

### Analysis of VLP production.

COS-1 or 293T cells were transfected as described above. Cells and supernatants were collected at 38 h posttransfection for COS-1 cells and at 15 to 20 h posttransfection for 293T cells. Cell lysates were harvested in 1× Triton-X buffer with freshly prepared protease inhibitor cocktail (Sigma) and RNaseOUT (Invitrogen). For collection of VLPs, supernatants were centrifuged at 1,000 rpm (225 × *g*) for 10 min at 4°C, filtered (0.45 μm) to remove remaining cells, and purified through a 30% sucrose cushion in an SW60Ti rotor at 60,000 rpm (370,000 × *g*) for 30 min at 4°C, as described previously (30).

### Velocity sedimentation.

Cells were harvested at 36 to 38 h posttransfection, in 1× NP-40 buffer (10 mM Tris acetate, pH 7.4, 50 mM KCl, 100 mM NaCl, 0.625% NP-40, 10 mM EDTA). For each sample, equivalent amounts of lysate were layered on a step gradient (10%, 15%, 40%, 50%, 60%, 70%, and 80% sucrose in lysis buffer) and subjected to velocity sedimentation in a Beckman MLS50 rotor at 45,000 rpm (162,500 × *g*) for 45 min at 4°C, as described previously (30). Gradients were fractioned from top to bottom.

### Membrane flotation.

Transfected cells were collected at 15 h (see Fig. 6A) or 36 h (see Fig. 6B) posttransfection, and cells were lysed using a 1-ml Dounce homogenizer in hypotonic lysis buffer (10 mM Tris acetate, pH 7.4), followed by addition of buffer after lysis to bring the final salt concentrations to 10 mM Tris acetate (pH 7.4), 50 mM KCl, 100 mM NaCl. Nuclei were removed by centrifugation at 225 × *g* for 10 min at 4°C. Postnuclear supernatants were mixed with 87% sucrose to a final concentration of ∼75% sucrose and used as the bottom layer of a 5-ml membrane flotation sucrose gradient, with 65% and 10% sucrose layered on top. Sucrose solutions were made in buffers with standard salts, described above, or with NaCl at a final concentration ranging from 0.25 M to 0.375 M (high-salt conditions). Flotation was performed by centrifugation in a Beckman MLS50 rotor at 35,000 rpm (98,400 × *g*), for 4 h at 4°C. Twelve fractions were collected starting from the top of the gradient, along with an additional 13th fraction that consisted of any denatured protein in the pellet. For experiments involving subsequent velocity sedimentation analyses of selected fractions, the membrane (M) fractions (fractions 3 and 4) were immediately pooled, and NP-40 was added to a final concentration of 0.625% followed by rotation for 30 min at 4°C. Subsequently, the M fraction was diluted to decrease the sucrose percentage in the sample below 10%, and equivalent amounts of the M fraction (∼3% of the total M fraction) were loaded onto separate velocity sedimentation gradients, as described above.

### Reverse transcription-quantitative PCR (RT-qPCR).

Transfected 293T cells were harvested ∼15 h posttransfection in 1× NP-40 buffer as described under “Transfection, immunoprecipitations, and Western blot analyses” above. Cell lysate containing 20 μg of cellular protein was subjected to immunoprecipitation with antibody to ABCE1 or nonimmune rabbit IgG, as described above. Immunoprecipitations and inputs were used either for WB analysis, as described above, or for RNA extraction using TRIzol (Invitrogen) followed by precipitation with isopropanol. RNA was resuspended in 50 μl of DNase buffer and treated with DNase I (Invitrogen). To generate cDNAs, reaction mixtures were programmed with 5 μl of RNA using random hexamers as primers and reverse transcribed (Superscript VILO cDNA synthesis kit; Invitrogen) followed by treatment with RNase H (Bio-Rad). For every sample, a negative control lacking master mix (RT-minus) was analyzed in parallel, and every experiment included a nontemplate control. A beads-alone control was used to determine background HIV-1 RNA levels. HIV-1 genomic RNA was quantified via qPCRs performed in duplicate, programmed with 2 μl of diluted cDNA and oligonucleotides (F, 5′-AGAAGGCTGTAGACAAATACTGGG-3′; R, 5′-TGATGCACACAATAGAGGGTTG-3′) which amplified a 108-bp product between bp 162 and 270 of the Gag open reading frame in HIV-1, LAI strain. In parallel, a standard curve was generated using serial 10-fold dilutions of a Gag amplicon that was previously analyzed using PicoGreen to quantify the number of DNA copies per microliter in the immunoprecipitation samples. qPCR products were detected with the iQ SYBR green Supermix (Bio-Rad) and analyzed using the Bio-Rad MyiQ reverse transcription-PCR (RT-PCR) detection system and iQ5 software (Bio-Rad).

### Preparation of virus for immunolabeling experiments.

Vesicular stomatitis virus glycoprotein (VSV-G)-pseudotyped VLPs were generated by transfecting 293T cells with HIV-1-GFPΔEnv, a second-generation Gag-Pol-containing packaging vector (psPAX2), and VSV-G (pMD2.G) at a ratio of 3:2:1. The latter two plasmids were obtained from Didier Trono (Swiss Institutes of Technology, Lausanne, Switzerland). At 6 h posttransfection, cells were washed three times with phosphate-buffered saline (PBS) and replaced with complete medium. To harvest virus, supernatants were collected at 48 h and 72 h posttransfection, pooled, centrifuged at 900 × *g* for 10 min at 4°C, and filtered (0.45 μm) to remove cells. Virus was pelleted by centrifugation in a Beckman SW40Ti rotor at 19,900 rpm (50,000 × *g*) for 90 min at 20°C, and infectivity was measured on TZM-bl cells (from Tranzyme Inc. through the NIH AIDS Research and Reference Reagent Program, Division of AIDS, NIAID, NIH). Infection of TZM-bl cells was facilitated by the addition of 20 μg/ml DEAE-dextran and by spinoculation in a Beckman GH3.8 rotor at 2,500 rpm (1,015 × *g*) for 2 h at 20°C.

### Immunogold labeling, electron microscopy, and quantitation.

COS-1 cells were infected with VSV-G-pseudotyped virus at a multiplicity of infection (MOI) of 40 to 50 using 20 μg/ml DEAE-dextran and spinoculation, as described above. Following incubation with virus for 12 to 15 h, cells were washed 3 times with medium to remove virus inoculum. Infected cells were harvested at 36 h postinfection in fixative (3% paraformaldehyde, 0.025% glutaraldehyde in 0.1 M phosphate buffer, pH 7.4), pelleted, and subjected to high-pressure freezing using the Leica EMPACT2, followed by freeze substitution. Samples were infiltrated overnight with LR White embedding resin (London Resin Company Ltd., Reading, Berkshire, England) in ethanol, changed to straight LR White, embedded in gelatin capsules (Electron Microscopy Sciences [EMS], Hatfield, PA, USA), and cured overnight in a UV light cryochamber at 4°C. Sections (∼50 nm) were placed on grids, treated with 0.05 M glycine for 20 min at room temperature, rinsed in PBS, blocked for 45 min with 1% bovine serum albumin (EMS), and washed in PBS with 0.1% bovine serum albumin C (BSA-C) (EMS). For immunogold labeling, peptide-specific antisera directed against ABCE1 (33) were affinity purified, desalted, and concentrated to 1 to 2 mg/ml. Grids were double labeled with ABCE1 and Gag. Labeling with anti-ABCE1 (0.7 mg/ml in 0.1% BSA-C with 0.001% Tween 20) was detected using goat anti-rabbit F(ab′)_2_ fragment conjugated to 15-nm gold particles (EMS). Grids were subsequently labeled with a murine monoclonal antibody directed against HIV-1 Gag p24 (hybridoma 183-H12-5C) (0.125 mg/ml in 0.1% BSA-C), which was detected using goat anti-mouse F(ab′)_2_ fragment conjugated to 6-nm gold particles (EMS). Fixation, staining, imaging with the JEOL-1400 transmission electron microscope, and image acquisition have been described previously (32). Each immunolabeling experiment included two sections from each of four groups (wild-type [WT] HIV-1 and the Q155N, ΔMHR, and G2A mutants).

To quantify the relative amount of PM targeting and the stages of assembly at the PM, images of WT Gag and the ΔMHR and G2A mutants from two independent immunoelectron microscopy (immuno-EM) labeling experiments were analyzed quantitatively. For each of the three groups in a single experiment, three cells were identified randomly, as described previously (32), and photographs were taken to encompass the perimeter of each cell (approximately 10 photographs per cell, with a total of 6 cells quantified for each group when the two experiments were combined). A Gag cluster was defined as three Gag labels within a 100-nm diameter. All Gag clusters within 100 nm of the PM in these images were quantified. To be defined as an independent cluster, the center of a Gag cluster needed to be separated by 100 nm from the center of an adjacent Gag cluster. The total number of PM Gag clusters and the length of PM analyzed are shown in Table 1. Gag clusters were categorized either as targeted Gag, an early assembly site, or a late assembly site. Definitions of targeted Gag, early assembly sites, late assembly sites, and Gag-ABCE1 colocalization are provided in Table 1, along with the total number of PM Gag clusters analyzed and the total length of PM analyzed. Each experiment was quantified separately, and the average ± standard error of the mean (SEM) from the two experiments was determined (*n* = 2). After quantitation was completed, images were chosen for Fig. 7 that together illustrate the approximate distribution of targeted, early, and late sites quantified, as shown in Table 1 and Fig. 8.

**TABLE 1:** Immuno-EM quantitation

Proviral construct	Total PM length analyzed (μm)	Total no. of Gag clusters analyzed	Total no. of Gag clusters per 10 μm of PM ± SEM	No. of targeted Gag clusters^*a*^ per 10 μm of PM ± SEM (% of total)	No. of early assembly sites^*b*^ per 10 μm of PM ± SEM (% of total)	No. of late assembly sites^*c*^ per 10 μm of PM ± SEM (% of total)	No. of targeted Gag clusters colocalized with ABCE1^*d*^ per 10 μm of PM ± SEM (% of total targeted)	No. of early assembly sites colocalized with ABCE1^*d*^ per 10 μm of PM ± SEM (% of total early)	No. of late assembly sites colocalized with ABCE1^*d*^ per 10 μm of PM ± SEM (% of total late)
WT	91.8	246	26.3^*e*^ ± 7.5	9.4 ± 2.2 (36)	10.1 ± 2.0 (38)	6.8 ± 3.3 (26)	2.1^*g*^ ± 0.7 (22)	4.0 ± 0.01 (40)	2.9 ± 2.0 (43)
ΔMHR	97.7	315	32.2^*e*^^,^^*f*^ ± 0.9	32.2 ± 1.0 (100)	0.1 ± 0.1 (0)	0 (0)	5.0^*g*^ ± 1.6 (15)	0.1 ± 0.1 (100)	0 (0)
G2A	87.5	60	6.9^*f*^ ± 0.5	6.3 ± 0.8 (92)	0.5 ± 0.5 (7)	0.1 ± 0.1 (2)	0.5 ± 0.5 (7)	0 (0)	0.1 ± 0.1 (100)

a Targeted Gag was defined as a Gag cluster without obvious curvature of the PM.

b Early assembly site was defined as a Gag cluster with <50% curvature of the PM.

c Late assembly site was defined as a Gag cluster with ≥50% curvature of the PM.

d ABCE1 colocalization was defined as ABCE1 labeling within a 170-nm diameter from the center of a Gag cluster.

e These two values were not significantly different from each other (*P* >0.5).

f These two values were significantly different from each other (*P* <0.002).

g These two values were not significantly different from each other (*P* >0.5).

## RESULTS

### MHR deletion or conserved residue point mutations reduce HIV-1 particle production in 293T and COS-1 cells.

**FIG 2:**
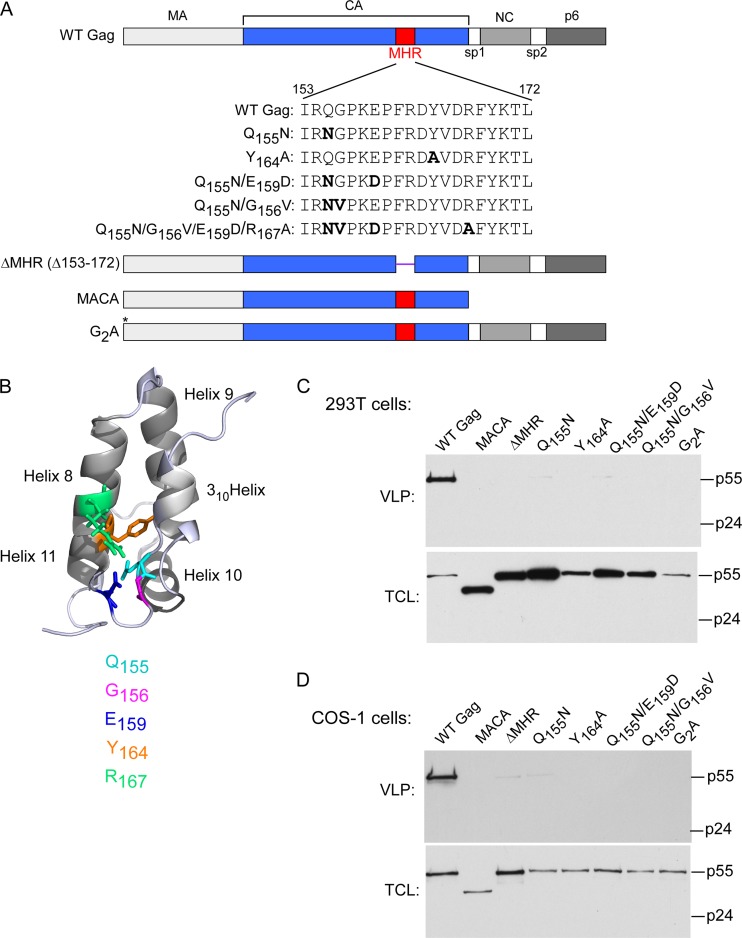
HIV-1 proviruses expressing an MHR deletion or conserved residue mutations fail to form VLPs. (A) Diagram of HIV-1 proviral Gag proteins (LAI strain) analyzed in this study showing WT Gag domains (MA, CA, NC, and p6) and spacer peptides (sp1 and sp2), with CA in blue and the MHR in red. Amino acid sequences from the MHR region of WT Gag (residues 153 to 172 in CA) and MHR point mutants studied here are shown, with mutated residues in bold, deleted residues in parentheses, and an asterisk showing the location of the G2A mutation. (B) High-resolution structure of CA-CTD (PDB accession number 2KOD [48]) showing side chains of the five invariant or highly conserved MHR residues studied here shown in the colors indicated. (C and D) 293T cells (C) or COS-1 cells (D) were transfected with proviruses expressing WT Gag or Gag containing the indicated MHR deletion or mutations. VLPs and total cell lysates (TCL) were harvested and analyzed by WB assay for Gag, with migrations of full-length Gag (p55) and CA (p24) indicated. Data in each panel are from a single experiment that is representative of two independent experiments.

In the case of HIV-1, the MHR precedes and overlaps with the CA helix 8, which is located at the N terminus of the CA-CTD subdomain (Fig. 2A). Based on the alignment in Fig. 1A, the consensus sequence for the MHR is Q(G/K)X_2_EX_4/5_(Y/F)X_2_(R/G)(F/L)X_3_H, with X being any residue and H being a hydrophobic residue. In this 19-amino-acid consensus, there are two invariant residues (Q1 and E5) and four highly conserved residues (G/K2, Y/F11, R/G14, and F/L15). Here, we studied deletion of the HIV-1 MHR as well as mutations of the two invariant and three highly conserved HIV-1 MHR residues (Fig. 1A and 2A) (HIV-1 Q155 and E159 are invariant; G156, Y164, and R167 are highly conserved). Notably, four of the five residues that we studied are part of a hydrogen bond network in the HIV-1 CA-CTD crystal structure (8, 9) (Q155, G156, E159, and R167 [Fig. 2B]).

Others have shown that point mutations of highly conserved residues in the N terminus of the HIV-1 MHR cause dramatic reductions in VLP production without affecting intracellular steady-state levels of Gag (11, 13). In those studies, HIV-1 VLP production was inhibited by conservative substitutions in the MHR invariant residues (Q155N and E159D), a nonconservative substitution of a highly conserved residue (Y164A), or, in one case, an MHR deletion. Those studies also established that the same VLP reduction is observed in both a WT and a *pro*-minus context (in which the HIV-1 protease was mutated or absent [11, 13]). Thus, earlier studies demonstrated that HIV-1 MHR mutations consistently block assembly at some point prior to completion of the immature Gag lattice, although, as described above, no consensus was reached about the timing of this block.

Here, we first confirmed and extended the previous VLP inhibition findings by expressing HIV-1 subtype B *pro*-minus proviruses containing point mutations in a variety of conserved MHR residues or an MHR deletion (Fig. 2A), in two different cell types. The *pro*-minus context was utilized so that Gag could be quantified easily. We found that single point mutations of the invariant MHR residues, Q155 and E159, inhibited VLP production without significantly affecting steady-state intracellular Gag levels in transfected 293T (human) or COS-1 (African green monkey) cells (Fig. 2C and D). Similar results were observed upon mutation of the highly conserved Y164 residue, deletion of the entire MHR (ΔMHR), or mutation of two highly conserved or invariant MHR residues (Q155N/E159D and Q155N/G156V). Two other mutations known to inhibit VLP production (reviewed in reference 1; MACA Gag [ΔNC/p6] and G2A Gag [which fails to undergo myristoylation]) were analyzed in parallel as negative controls.

### Like wild-type Gag, MHR mutants associate with the host proteins ABCE1 and DDX6.

**FIG 3:**
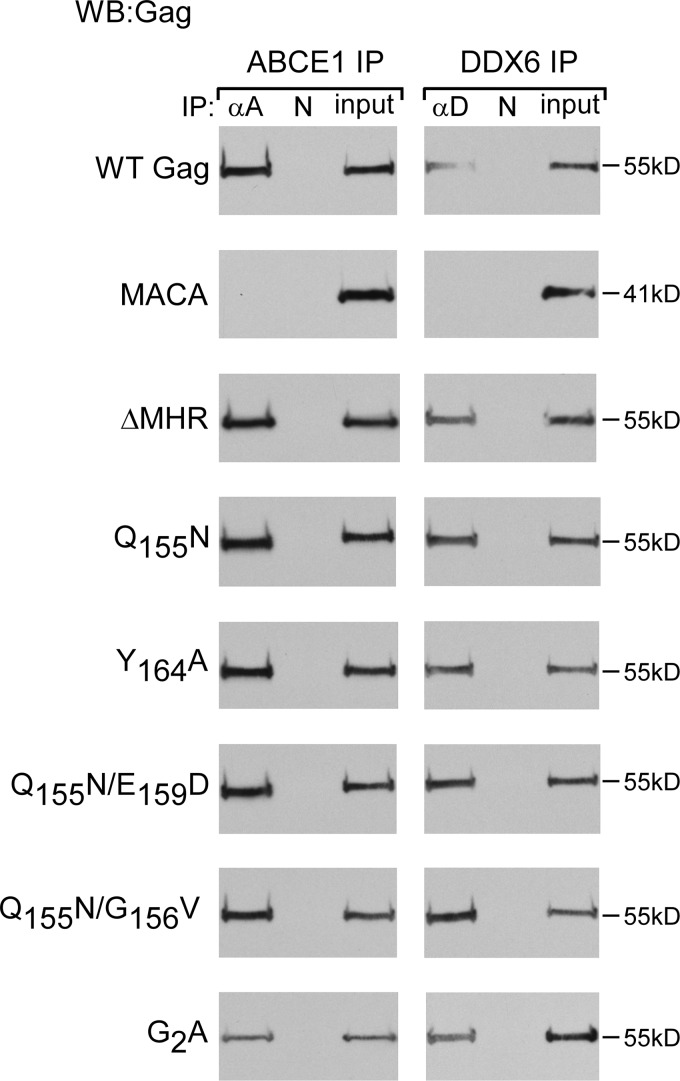
Assembly-defective MHR mutants associate with ABCE1 and DDX6. Human 293T cells were transfected with the indicated WT or mutant provirus. Lysates were immunoprecipitated with antiserum to ABCE1 (αA), antiserum to DDX6 (αD), or nonimmune IgG (N), followed by WB analysis for Gag. Equivalent inputs were also analyzed for Gag by WB analysis. Data are from a single experiment that is representative of three independent experiments.

Next, we examined whether the MHR deletion or conserved residue mutations inhibit association of HIV-1 Gag with the cellular facilitators of assembly ABCE1 and DDX6 (Fig. 3), which are markers for formation of high-molecular-weight cytosolic and membrane-bound assembly intermediates (27, 29, 31, 32, 37). Immunoprecipitation of 293T cell lysates expressing the wild-type (WT) or mutant HIV-1 provirus with antibody to ABCE1 or DDX6 followed by immunoblotting revealed that Gag proteins containing an MHR deletion or conserved MHR residue point mutations are associated with ABCE1 and DDX6, unlike the MACA Gag negative control (Fig. 3) (27, 29, 31, 32, 37). These data indicated that MHR deletion or conserved MHR residue mutations do not inhibit recruitment of Gag into the previously described precursor RNA granule complex that contains ABCE1 and DDX6 (32). Additionally, because we had previously demonstrated that ABCE1 and DDX6 are not associated with unassembled ∼10S Gag (29, 32), these findings suggested that these MHR mutants are likely not arrested as ∼10S assembly intermediates.

### MHR mutants are arrested at the ∼80S assembly intermediate at the plasma membrane.

**FIG 4:**
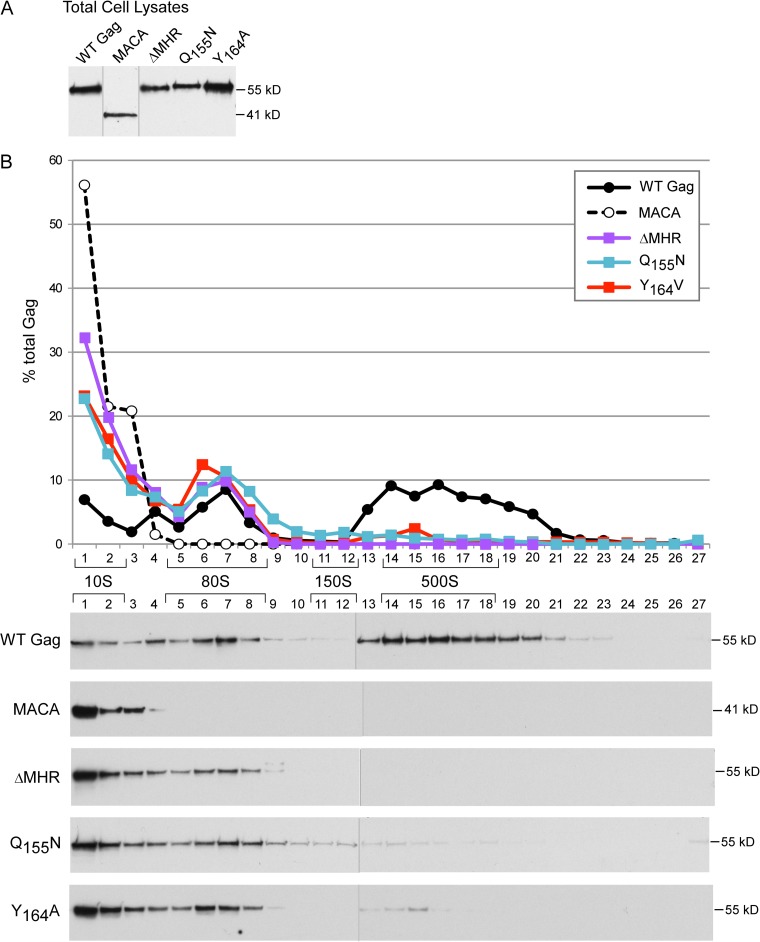
Single point mutations of highly conserved MHR residues or MHR deletion arrest assembling Gag at the ∼80S intermediate. COS-1 cells were transfected with the indicated WT or mutant provirus. Equivalent aliquots of cell lysates were analyzed by WB assay for Gag (A) or subjected to velocity sedimentation, with gradient fractions analyzed by WB assay for Gag (B). The graph depicts relative amounts of Gag in each fraction of the blots below (as percentage of total Gag), with the approximate S value of each assembly intermediate indicated by brackets. Data are from a single experiment that is representative of three independent experiments.

**FIG 5:**
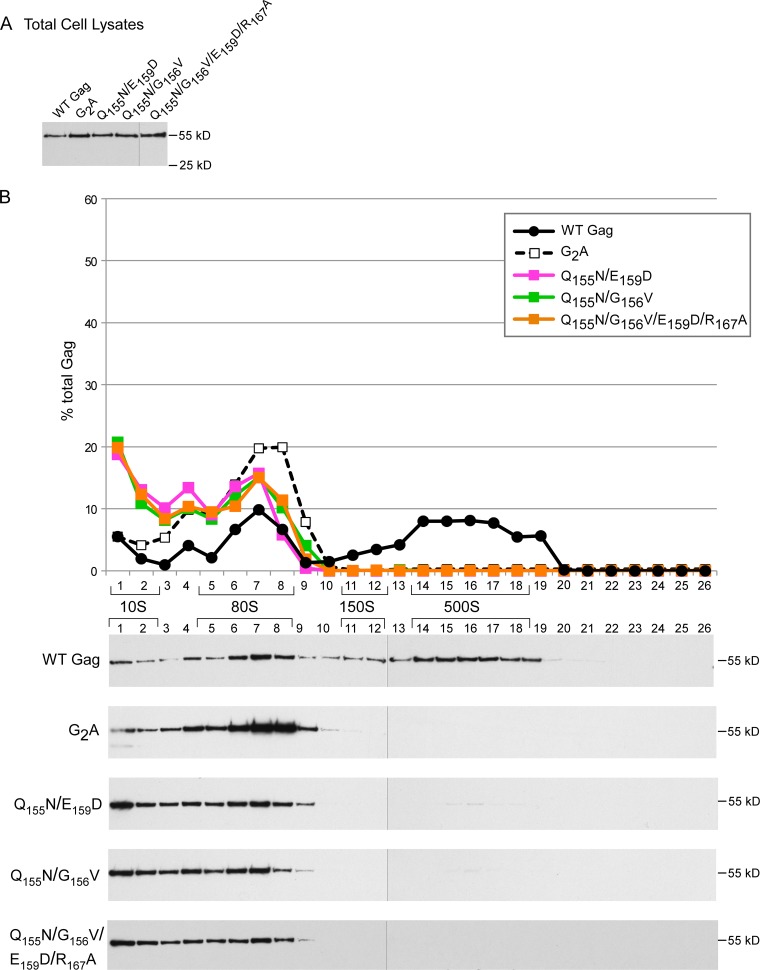
Double or quadruple MHR point mutations arrest assembling Gag at the ∼80S intermediate. 293T cells were transfected with the indicated WT or mutant provirus. Equivalent aliquots of cell lysates were analyzed by WB assay for Gag (A) or subjected to velocity sedimentation, with gradient fractions analyzed by WB assay for Gag (B). The graph depicts relative amounts of Gag in each fraction of the blots below (as percentage of total Gag), with the approximate S value of each assembly intermediate indicated by brackets. Data are from a single experiment that is representative of two independent experiments.

To further pinpoint which step in the assembly pathway is inhibited by these MHR mutations, we analyzed cell lysates expressing WT or mutated Gag proteins by velocity sedimentation under conditions in which steady-state expression levels of WT and mutant Gag proteins were comparable (Fig. 4 and 5). As shown previously (30–32), at steady state WT Gag was found in the ∼10S, ∼80S, and ∼500S assembly intermediates, with very little in the highly transient ∼150S complex. In contrast, ΔMHR and the single point mutations Q155N and Y164A all resulted in formation of the ∼10S and ∼80S intermediates, with little or no production of the ∼500S intermediate, in both COS-1 (Fig. 4) and 293T (data not shown) cells. We also asked whether mutations of additional invariant (E159) or highly conserved (G156) residues caused the same arrest as that observed for the single point mutants or caused an earlier arrest with formation of exclusively the ∼10S complex. Analysis of two double point mutants, Q155N/E159D and Q155N/G156V, both in 293T cells (Fig. 5) and in COS-1 cells (data not shown), and a quadruple mutant involving substitution of both invariant residues (Q155 and E159) and two highly conserved residues (G156 and R167) revealed an ∼80S arrest phenotype (Fig. 5). Thus, MHR deletion and single, double, or quadruple point mutations of highly conserved MHR residues all appear to block progression past the ∼80S intermediate. Note that because these analyses were performed under steady-state conditions, it is not surprising that both the ∼10S and ∼80S intermediates are observed when there is an ∼80S arrest, since newly synthesized Gag that has recently entered the pathway will be present in the ∼10S form. Notably, the amount of ∼10S Gag was three to four times higher for the MHR mutants than for WT Gag (Fig. 4 and 5), as will be discussed below.

**FIG 6:**
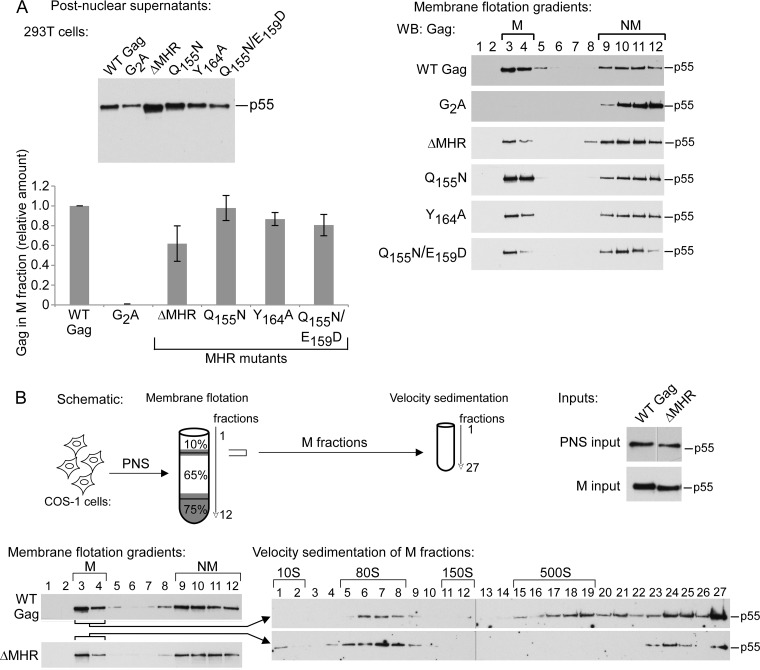
MHR mutants target to membranes but fail to form the ∼500S membrane-bound intermediate. (A) 293T cells expressing the indicated WT and mutant proviruses were subjected to hypotonic lysis. Postnuclear supernatants were analyzed by WB assay for Gag (left) and subjected to membrane flotation followed by WB assay for Gag (right). Membrane (M) and nonmembrane (NM) fractions are indicated. The graph shows the relative amount of Gag in the membrane fraction, with the percentage of WT Gag in the membrane fraction (55.3%) set to 1. Data in the graph represent an average from two independent experiments ± SEM, with blots from a single representative experiment. (B) COS-1 cells expressing the indicated proviruses were harvested for membrane flotation followed by velocity sedimentation, as shown in the schematic. Inputs show WB analysis of Gag in equivalent aliquots of postnuclear supernatants (PNS) and membrane fractions (M). Membrane flotation and velocity sedimentation fractions were also analyzed by WB assay for Gag. Data are shown for COS-1 cells transfected with WT and ΔMHR proviruses, but similar results were also obtained for Q155N and Y164A (data not shown). Gag observed in fractions 24 to 27 likely represents denatured aggregates of Gag that are produced following repeated centrifugation of lysates, as described previously (31). Data are from a single experiment that is representative of two independent experiments.

Next, we asked whether the arrested ∼80S intermediates formed by the MHR mutants are in the cytosol or at the PM. Previously, we had shown that both the largest WT Gag complex in the cytosolic fraction and the smallest WT Gag complex in the membrane fraction migrate at ∼80S when solubilized with nonionic detergent to remove associated lipids and analyzed by velocity sedimentation (31). The simplest interpretation for this observation is that the ∼80S intermediate transports Gag from the cytosol to the PM, as shown in Fig. 1B. To determine if the MHR mutants are arrested before or after membrane targeting of the critical ∼80S intermediate, we performed a membrane flotation analysis on 293T cells expressing comparable steady-state levels of WT and mutant Gag (Fig. 6A). The amount of WT Gag found in the membrane (versus nonmembrane) fraction at steady state in different experiments ranged from 48 to 63% (average of 55% [data not shown]). We found only a minimal reduction in membrane targeting of the MHR mutants, with the amount of membrane targeting relative to WT being 98% for Q155N, 86% for Y164A, 81% for the Q155N/E159D double mutant, and 62% for the ΔMHR mutants (Fig. 6A, graph). Similar results were obtained for Q155N/G156V and the quadruple point mutant (data not shown). Further analysis of membrane fractions by velocity sedimentation confirmed that membrane-associated Gag produced by the Q155N, Y164A, and ΔMHR mutants contained only the ∼80S intermediate, but no ∼500S intermediate, while membrane fractions of WT Gag contained both the ∼80S and ∼500S intermediates (Fig. 6B and data not shown).

**FIG 7:**
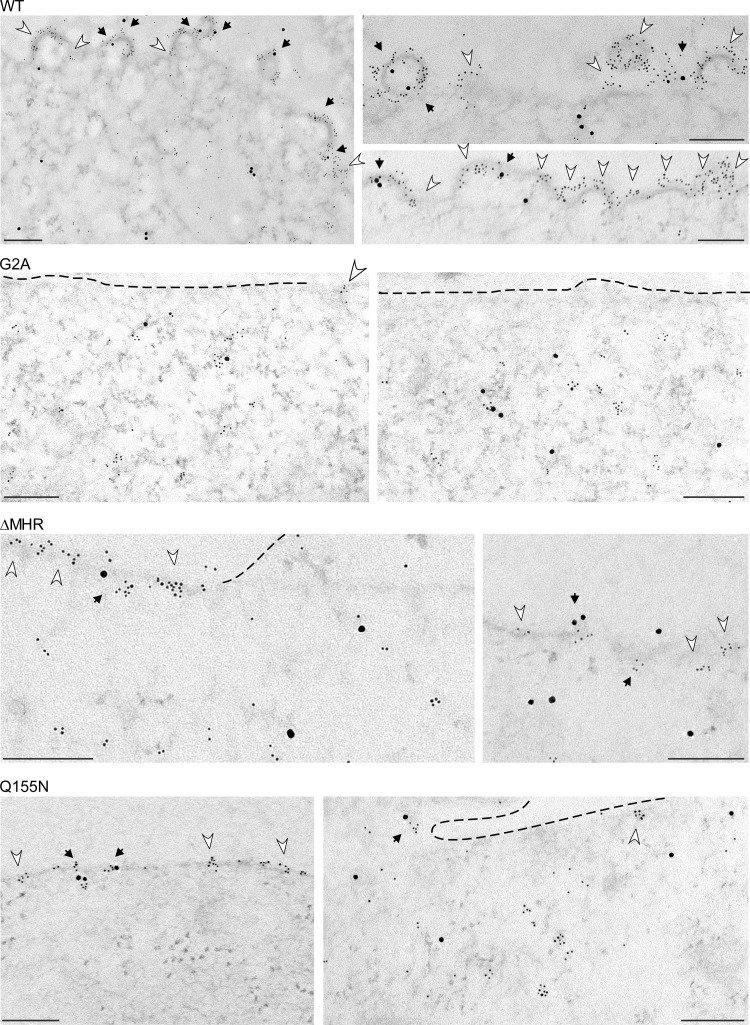
Immunoelectron micrographs of MHR mutants arrested as assembly intermediates at the PM. COS-1 cells infected with proviruses expressing WT Gag or the indicated mutants were analyzed by double-label immuno-EM, using antibodies to Gag and ABCE1. Small gold particles indicate Gag labeling; large gold particles indicate ABCE1 labeling. Images were chosen from the quantitative immuno-EM experiments (Fig. 8 and Table 1) and represent the approximate distribution of targeted Gag, early assembly, and late assembly sites at the PM observed for each group. Dark arrows show Gag clusters at the PM that are colocalized with ABCE1; white arrowheads show Gag clusters at the PM that are not colocalized with ABCE1. Dashed lines are placed in some locations to help viewers identify the PM. Bars, 200 nm. As described previously (30), not all instances of colocalization are captured by immuno-EM since the 50-nm sections capture only a fraction of the ∼100-nm-diameter capsid. Targeted Gag, early assembly sites, and late assembly sites are defined in the text and in Table 1.

**FIG 8:**
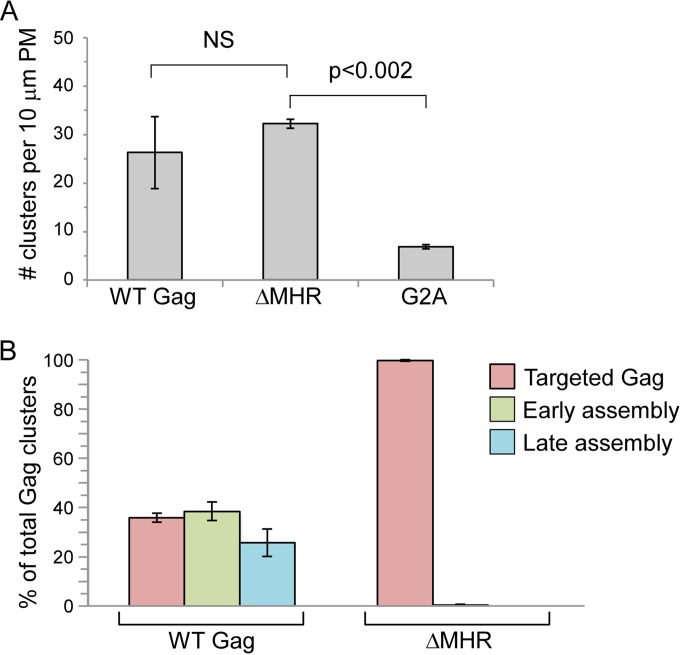
Quantitative immuno-EM confirms that MHR mutations arrest Gag after PM targeting. Immuno-EM images of infected COS-1 cells described in Fig. 7 were analyzed quantitatively (Table 1). For the indicated constructs, graphs show the average number of Gag clusters in 10 μm of PM (A) and the number of targeted Gag clusters, early assembly sites, and late assembly sites at the PM as a percentage of total Gag clusters at the PM (B). Targeted, early, and late sites are defined in the text and in Table 1. Error bars show standard errors of the means from two independent experiments. Brackets indicate two comparisons that were either not significant (NS) or significant (*P* < 0.002) using the unpaired Student *t* test.

Thus, when taken together, our membrane flotation, coimmunoprecipitation, and velocity sedimentation data suggest that all the MHR mutations that we had analyzed—including single conserved MHR residue mutations, multiple conserved MHR residue mutations, and MHR deletion—cause assembling Gag to be arrested as a membrane-associated ∼80S intermediate. To confirm these findings, we analyzed COS-1 cells infected with virus expressing WT or mutated Gag using immunoelectron microscopy (immuno-EM) with double labeling of Gag and ABCE1 using small and large gold, respectively (Fig. 7 and 8; Table 1). Images were quantified to determine the average number of Gag clusters per 10 μm of PM length (Fig. 8; Table 1). Additionally, we quantified the number of Gag clusters that were in the form of targeted Gag (in which Gag clusters are at the PM but cause no membrane deformation), early stages of assembly (in which Gag clusters are at a budding site that deforms the membrane but displays <50% of full curvature), and late stages of assembly (in which Gag clusters are at a budding site that displays ≥50% of full curvature). Finally, the number of Gag clusters in each category that were associated with ABCE1 was also quantified (Table 1).

As shown previously (32), clusters of WT Gag were abundant at the PM (26.3 per 10 μm of PM length) and were distributed relatively evenly between targeted Gag and early and late stages of assembly (Fig. 7 and 8; Table 1). Considerably fewer G2A Gag clusters were found at the PM (6.9 per 10 μm of PM length [Fig. 7 and 8; Table 1]), which was as expected given that G2A is defective in membrane targeting. Notably, ΔMHR formed abundant Gag clusters at the PM (32.2 per 10 μm of PM length). The number of ΔMHR clusters at the PM was not significantly different from the number of WT Gag clusters at the PM (*P* > 0.5) (Fig. 8; Table 1) but was significantly greater than the number of G2A clusters at the PM (*P* < 0.002) (Fig. 8; Table 1). Thus, the results of quantitative EM analysis are generally consistent with the results of the membrane flotation analysis in Fig. 6. Additionally, the quantitative EM analysis revealed that 100% of ΔMHR Gag clusters at the PM membrane are in the form of targeted Gag, which lacks any of the membrane deformation characteristic of early or late assembly. In contrast, only 36% of WT Gag clusters are in the form of targeted Gag, with the remaining 64% of WT Gag clusters exhibiting membrane deformation characteristic of either early (38%) or late (26%) assembly sites (Fig. 7 and 8; Table 1). Similar results were observed upon immuno-EM analysis of Q155N (Fig. 7 and data not shown). We also observed that 22 to 43% of WT Gag clusters at the PM in cells exhibited double labeling for Gag and ABCE1 (with the exact amount of colocalization depending on the stage of assembly) (Fig. 7; Table 1), in agreement with previously published immuno-EM results (31, 32). Consistent with our coimmunoprecipitation results (Fig. 3), clusters of ΔMHR and Q155N Gag were also colabeled with antibody to ABCE1 in immuno-EM analyses (Fig. 7; Table 1; also data not shown), albeit to a somewhat lesser extent (15% for targeted ΔMHR clusters versus 22% for targeted WT Gag clusters [Table 1]). Additionally, G2A appeared to form clusters in the cytoplasm that were frequently colabeled with ABCE1 (Fig. 7). Thus, the immuno-EM analyses confirm the conclusion from biochemical studies that MHR deletion or conserved residue mutations arrest assembling HIV-1 Gag as an ∼80S membrane-bound assembly intermediate that is associated with the cellular facilitator ABCE1.

### The arrested ∼80S assembly intermediates formed by some MHR mutants are unstable upon high-salt treatment.

**FIG 9:**
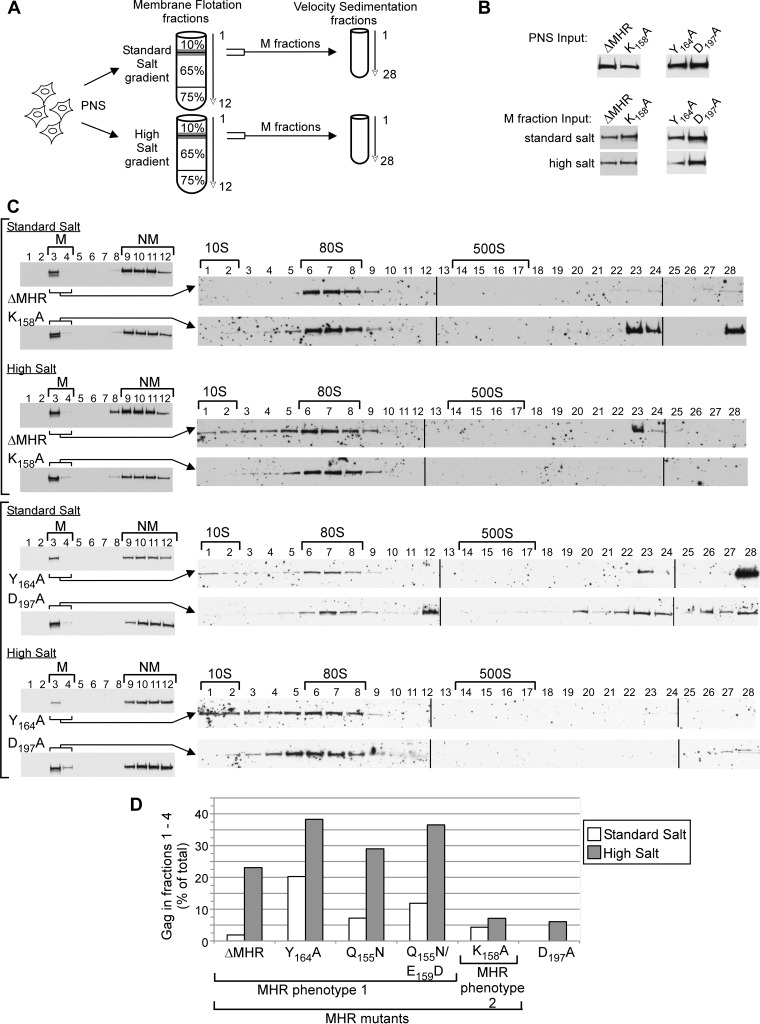
Some MHR mutants are arrested as a labile ∼80S assembly intermediate. (A) Schematic showing that COS-1 cells expressing the indicated WT and mutant proviruses were analyzed by membrane flotation in the presence of standard intracellular (0.1 M NaCl) or high (0.25 to 0.375 M NaCl) salt concentrations; subsequently, membrane fractions (M fractions) were further analyzed by velocity sedimentation. The D197A and K158A mutants served as controls since they were previously found to be stable upon high-salt treatment (31). (B) Blots show membrane flotation fractions analyzed by Western blot (WB) assay for Gag, with membrane (M) and nonmembrane (NM) fractions indicated. PNS, postnuclear supernatant. (C) Shown are fractions analyzed by Western blotting for Gag from two experiments, with each experiment enclosed by vertical brackets. To the left are membrane flotation blots performed under standard or high-salt conditions, with M fractions and nonmembrane (NM) fractions indicated by horizontal brackets. To the right are blots from velocity sedimentation analyses of M fractions. Approximate S values are shown with horizontal brackets above blots. (D) Graph of the amount of Gag (as percentage of total Gag) present in small complexes (≤60S, i.e., fractions 1 to 4) in velocity sedimentation analyses of membrane fractions isolated under standard-salt or high-salt conditions. Western blots of inputs for the graphed constructs are shown in panel B; Western blots for Q155N and Q155N/E159D are not shown. Note that the repeated ultracentrifugations required for this experiment resulted in precipitation of some Gag, most likely due to aggregation, observed most prominently in fractions 20 to 28 under standard-salt conditions, as noted previously (31). Graphed data are from a single experiment that is representative of 2 to 4 independent experiments.

Previously, we demonstrated that mutations in CA helix 9 result in arrest of Gag as a membrane-associated ∼80S assembly intermediate that is labile, in that it dissociates into smaller complexes upon treatment with high levels of salt (31). In contrast, mutations in the CA-CTD base region, including the K158 mutation in the MHR, result in arrest of Gag as a stable, membrane-associated, ∼80S intermediate that retains its integrity upon high-salt treatment, as does the ∼80S intermediate formed by WT Gag (31). Here, we performed a similar analysis of additional MHR mutants, in which membrane fractions were collected in the presence of standard or high levels of salt and then subjected to velocity sedimentation in the presence of nonionic detergent (Fig. 9). We found that the Y164A, Q155N, Q155N/E159D, and ΔMHR mutations cause arrest of Gag as a labile, membrane-associated ∼80S intermediate that loses its integrity upon high-salt treatment, as indicated by some Gag shifting from the ∼80S position into smaller complexes (≤60S, fractions 1 to 4) following high-salt treatment (Fig. 9D, with blots for selected constructs shown in Fig. 9C). This contrasts with the less conserved K158 MHR residue and the D197 residue located in CA helix 10 (not in the MHR), both of which were found to be stable upon high-salt treatment, here (Fig. 9C and D) and previously (31). Note that while some membrane-associated Gag isolated under standard-salt conditions formed denatured aggregates upon velocity sedimentation, little aggregation was observed when membrane-associated Gag isolated under high-salt conditions was analyzed by velocity sedimentation (Fig. 9C). Thus, almost all the membrane-associated K158A and D197A were in the form of the ∼80S intermediate after high-salt treatment (as was shown for WT Gag previously [31]); in contrast, membrane-associated ΔMHR and Y164A formed both ∼80S and ≤60S complexes after high-salt treatment. Interestingly, some Y164A is observed in the ∼10S position even after standard-salt treatment (Fig. 9C and D). This could be because Y164A is labile upon repeated spins even without exposure to high salt levels or because some of the Y164A ∼10S intermediate is membrane associated in cells at the start of the experiment. Nevertheless, quantitation of small complexes (≤60S, fractions 1 to 4) reveals that nearly twice as much membrane-associated Y164A is found in these small complexes upon high-salt treatment as upon standard-salt treatment, and all other mutants show even higher degrees of ∼80S dissociation upon high-salt treatment (Fig. 9D). Thus, together the data indicate that the conserved residue MHR point mutations and MHR deletion arrest Gag as a labile membrane-associated ∼80S assembly intermediate, in contrast to the K158A MHR mutant, which arrests Gag as a stable membrane-associated ∼80S assembly intermediate.

### The arrested ∼80S assembly intermediates resulting from MHR deletion or conserved residue MHR substitution contain gRNA.

**FIG 10:**
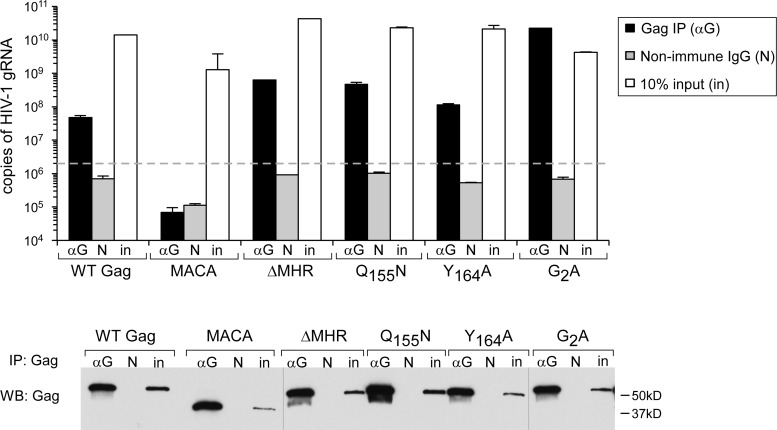
MHR deletion and point mutations of highly conserved MHR residues do not inhibit association of assembling Gag with HIV-1 genomic RNA. Total cell lysates from 293T cells expressing the indicated proviruses were subjected to immunoprecipitation (IP) with HIV immunoglobulin (αG) or a nonimmune control antibody (N). The graph shows the number of HIV-1 gRNA copies in immunoprecipitation eluates analyzed by RT-qPCR and in aliquots representing 10% of immunoprecipitation input (in). Also shown is a Western blot (WB) of immunoprecipitation eluates from total cell lysates, using HIV immunoglobulin, to confirm that αG immunoprecipitates Gag efficiently. Nonimmune antibody (N) and 5% of immunoprecipitation input are also shown in WB assays. The dashed line shows the limit of detection based on analysis of beads-alone control. Data in the graph are from a single experiment that is representative of three independent experiments. Error bars show standard deviations between duplicate samples.

Since others have shown that Gag first associates with gRNA in the cytoplasm (38), and the MHR mutants analyzed here were arrested as membrane-bound ∼80S intermediates (Fig. 4 to 9), we would expect that the assembly defect for these mutants occurs after the Gag-gRNA association is initiated. Indeed, when complexes immunoprecipitated by HIV immunoglobulin were analyzed by reverse transcription followed by quantitative PCR (RT-qPCR), no defect was observed in the amount of gRNA coimmunoprecipitated with each of the MHR mutants analyzed, relative to WT (Fig. 10). The negative-control MACA Gag, which lacks the NC region critical for gRNA association, failed to associate with gRNA in this experiment, as expected. Thus, MHR deletion and conserved MHR residue mutations inhibit assembly after Gag has associated with HIV-1 gRNA.

### MHR deletion and conserved residue mutations display the same phenotype in a subtype C Gag.

**FIG 11:**
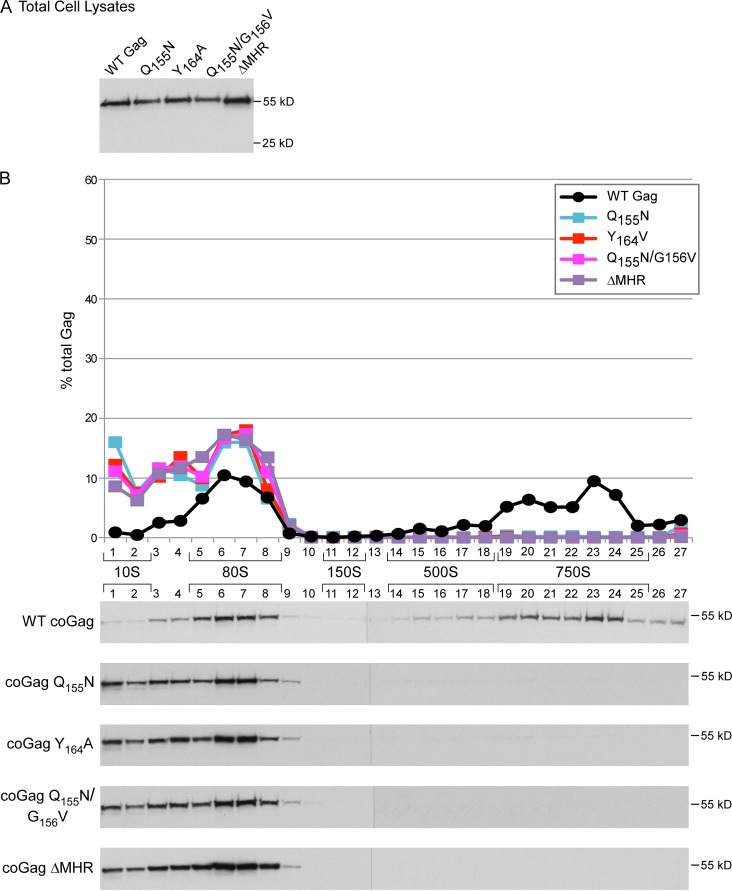
MHR mutations introduced into a codon-optimized, type C Gag from a primary isolate arrest assembly at the ∼80S intermediate. COS-1 cells were transfected with the indicated WT or mutant constructs encoding codon-optimized Gag from a subtype C primary isolate (36). Equivalent aliquots of cell lysates were analyzed by WB assay for Gag (A) or subjected to velocity sedimentation, with gradient fractions analyzed by WB assay for Gag (B). The graph depicts relative amounts of Gag in each fraction of the blots below (as percentage of total Gag), with the approximate S value of each assembly intermediate indicated by brackets. Data are from a single experiment that is representative of two independent experiments.

To this point, all the experiments in the current study utilized a lab-adapted, subtype B, HIV-1 isolate (LAI) in its native genomic context. However, primary HIV-1 isolates and other HIV-1 subtypes are more relevant to HIV-1 pathogenesis globally. Since the MHR is extremely well conserved, we would expect the same ∼80S arrest phenotype to be observed for Gags from primary isolates and other HIV-1 subtypes. To test this, we examined the effect of MHR deletion and conserved residue mutations on a Gag derived from a subtype C primary isolate (Fig. 11) (36). This Gag plasmid had also been codon optimized, which renders it independent of Rev-1 expression and independent of the CRM1 nuclear export machinery (39). Following nuclear export, wild-type codon-optimized Gag (coGag) is able to assemble and produce virus in human cells; however, the finding that coGag overcomes defects in assembly in murine cells suggests that it may traffic within the cytoplasm and/or assemble somewhat differently than native Gag, at least in some cell types (40). The amino acid sequence of the wild-type, subtype C, primary isolate that was used to make this plasmid is 81% identical and 90% similar to that of the subtype B lab-adapted Gag utilized in earlier figures and contained an MHR that was identical except for F instead of Y in position 16 (which is not a conserved residue [Fig. 1A]).

As observed for proviral subtype B Gag in Fig. 2 and 3, codon-optimized MHR mutants failed to produce VLPs (data not shown) but associated with ABCE1 to the same extent as WT by coimmunoprecipitation (data not shown). Moreover, codon-optimized, subtype C MHR mutants failed to progress past the ∼80S step, resulting in accumulation of the ∼10S and ∼80S assembly intermediates (compare Fig. 11 to 4 and 5). Thus, the phenotype of MHR deletion and conserved residue point mutations appears to be the same in both the subtype B, lab-adapted background and the subtype C, primary isolate amino acid context.

## DISCUSSION

**FIG 12:**
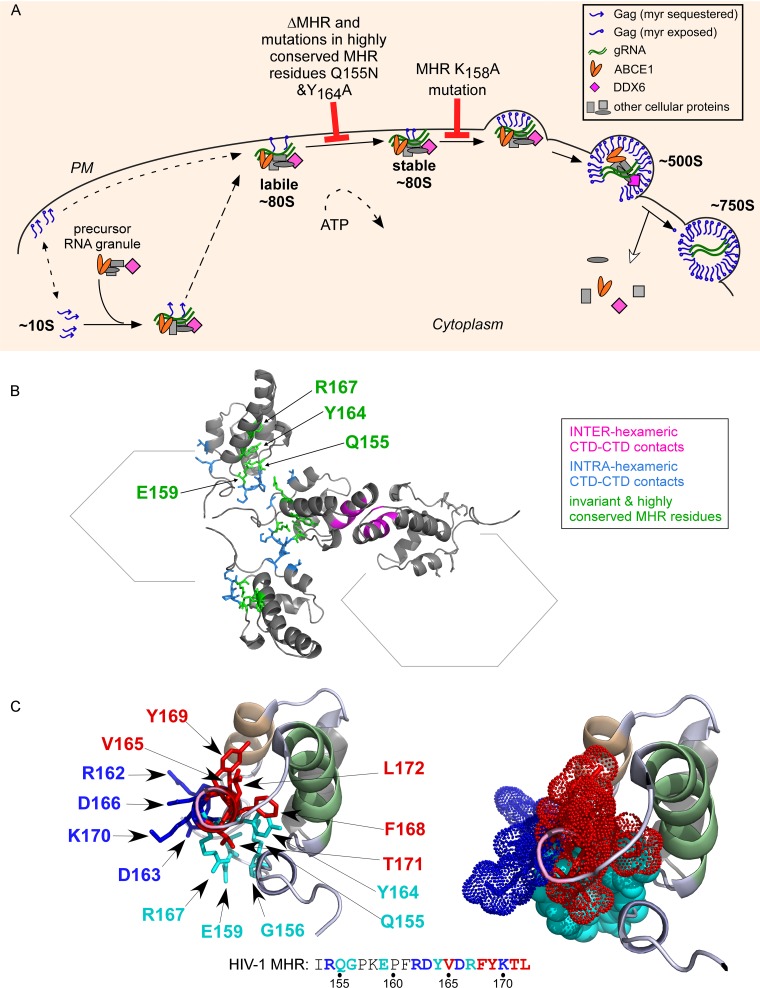
Model showing where specific MHR mutations arrest the HIV-1 assembly pathway. (A) Based on data presented here and elsewhere (10), MHR mutations inhibit assembly at one of two points following membrane targeting of an assembling Gag oligomer. Mutation of the highly conserved residues Y164 and Q155 or MHR deletion arrests Gag as a membrane-targeted ∼80S oligomer that is disrupted by high-salt treatment (labile ∼80S), while mutation of the less conserved K158 residue arrests Gag at the next step in the pathway as a stable membrane-targeted ∼80S oligomer. (B) To show the location of the MHR residues analyzed here relative to residues important for intrahexameric CA-CTD interfaces in the three-dimensional structure, we generated a ribbon diagram using a PDB file provided by J. Briggs, showing selected residues along with their side chains on three CA-CTD domains. Residues critical for the CA-CTD interhexa meric (dimer) interface are in pink (Q176, WMT184/185/186, and TL188/189), residues critical for intrahexameric CA-CTD contacts are in blue (G156, P157, K158, T218, and Q221), and MHR residues studied here are in green (Q155, Y164, R167, and E159). Note that G156 and K158 were also studied here but are shown in blue rather than green because they are known to form intrahexameric CA-CTD contacts (10). The ribbon diagram shows the predicted orientations of residues (but not relative distances) and reveals that MHR residues studied here are either in the intrahexameric CA-CTD interface or very close to it. (C) The structure on the left is the same high-resolution CA-CTD structure shown in Fig. 2B (PDB accession number 2KOD [48]) but this time rotated to display an end-on view of the MHR. Side chains for the five invariant or highly conserved MHR residues studied here are shown in turquoise and include both uncharged or polar residues (Y164, Q155, and G156) and charged residues (E159 and R167). Side chains for other MHR residues not studied here are also shown, including uncharged and polar residues in red (V165, F168, Y169, T171, and L172) and charged residues in blue (R162, D163, D166, and K170). The location of each residue in the amino acid sequence of the HIV-1 MHR is shown below using the same color coding. The structure on the right shows the same view and color coding but with side chains indicated by space-filling dotted clouds. CA helix 8 is shown in pink, CA helix 9 in green, and CA helix 11 in wheat.

Given that the MHR is required for assembly of HIV-1 and other retroviruses and is a highly conserved motif within the otherwise poorly conserved retroviral Gag protein, it is surprising that so little is known about when and how this motif acts during HIV-1 assembly. To better understand MHR function, we identified the exact stage at which conserved MHR residues are required during assembly of HIV-1 Gag, using quantitative biochemical and ultrastructural approaches. For our detailed analyses, we used subtype B HIV-1 proviruses containing either a deletion of the 18-amino-acid MHR motif or point mutations in one or more invariant or highly conserved residues in the MHR. We found that all of these MHR mutants associated with gRNA (Fig. 10) and with the cellular factors ABCE1 and DDX6 (Fig. 3). These findings argue that MHR mutants are not misfolded, consistent with an earlier study showing that coexpression of WT Gag can rescue assembly of MHR mutants (13). However, despite being capable of associating with gRNA and cellular proteins, Gag containing an MHR deletion or mutations of conserved MHR residues was unable to progress through the entire assembly pathway. Our analyses revealed that these mutants were arrested just after Gag had anchored to the PM but before it had formed a stable oligomer and before it started to form a viral bud (Fig. 4 to 9; summarized in Fig. 12A). The defect appeared to be the same whether one, two, or four invariant or conserved MHR residues were mutated (Fig. 5) and whether they were engineered into the subtype B provirus or a codon-optimized Gag whose amino acid sequence corresponds to a subtype C primary isolate (Fig. 11). Together, our findings demonstrate that the highly conserved MHR residues are not required for membrane targeting but are important for stabilization of the membrane-associated Gag oligomer.

Note that four of the techniques that we used here provide insight into the stability defect displayed by the membrane-targeted MHR deletion and conserved MHR residue mutants, with each technique revealing the defect to a different extent. At one extreme was immuno-EM, in which intact cells are fixed at the start of the harvest—one would not expect this technique to disrupt the labile, membrane-targeted MHR mutants, which would therefore be found at the PM (Fig. 7; Table 1). Along the same lines, while membrane flotation involves lysis of cells, this is done in the absence of detergent and therefore might not be expected to cause significant disruption of the labile, membrane-targeted MHR mutants (Fig. 6A). Thus, these two techniques are useful for demonstrating that MHR mutants are not defective in membrane targeting but provide little insight into the stability of the membrane-targeted complexes. In contrast, for velocity sedimentation cells are lysed in an isotonic buffer containing nonionic detergent—this technique would be expected to be somewhat disruptive to the labile, membrane-targeted MHR mutants and could therefore release some of the membrane-targeted population back into the cytosol. Such partial disruption of this labile, membrane-targeted population could explain why the amount of ∼10S Gag, which is largely cytosolic, is three to four times higher for MHR mutants than for WT Gag in velocity sedimentation analyses (Fig. 4), even though cytosolic levels do not appear higher for the MHR mutants than for WT Gag in the membrane flotation analyses (Fig. 6A) or by immuno-EM (Fig. 7). Finally, to intentionally disrupt the labile, membrane-targeted population, we used membrane flotation in high-salt buffer followed by velocity sedimentation in nonionic detergents (Fig. 9). This technique was the most effective for demonstrating that MHR deletion and conserved residue MHR mutants are in fact labile after membrane targeting, relative to WT Gag. Thus, the results of this spectrum of techniques are complementary and consistent, and together they confirm the two key findings of this study: that the MHR mutants target effectively to membranes but are less stable following membrane targeting than WT Gag.

Our conclusion that mutation of these invariant or highly conserved MHR residues inhibits assembly after membrane targeting of Gag differs from that of a previous study that found large defects in membrane targeting by MHR mutants (11). For example, in the earlier study, the amount of the Y164A mutant in the membrane fraction following membrane flotation was reduced to 38% of that observed for WT Gag, while we found that the amount of Y164 mutant in the membrane fraction was 87% relative to WT Gag (Fig. 6A). One possible explanation for the dramatic reduction in membrane targeting in the earlier study and the minimal to modest reduction observed in the current study is that assembly intermediates that are arrested at the PM may eventually undergo endocytosis followed by degradation. Such endocytic removal of arrested, membrane-associated assembly intermediates is likely to have progressed further at late times posttransfection, such as the 48-h time point used in the previous study (11), and may not be observed at the 15- to 38-h harvest times used in our study. Thus, analysis at late times posttransfection could miss the fact that initial membrane targeting was intact. Endocytosis of arrested membrane-associated ∼80S complexes could explain why large accumulations of the ∼80S intermediates are not observed for MHR mutants in velocity sedimentation gradients, in contrast to the G2A mutant, which forms large accumulations of the arrested cytosolic ∼80S intermediate (Fig. 4 and 5). As noted above, our conclusion that membrane targeting of MHR mutants is comparable to that of WT Gag was supported by our quantitative immuno-EM data (Fig. 8 and Table 1). Additionally, our findings are consistent with two previous analyses of a different MHR mutation in which an alanine substitution of the less conserved K158 residue resulted in a dramatic reduction in virus-like particle production, with Gag found at the PM in unassembled or aberrant structures (7, 31).

Our conclusion that MHR deletion inhibits HIV-1 assembly as dramatically as does substitution of highly conserved MHR residues is consistent with two other studies in which MHR deletions caused dramatic reductions in assembly (11, 41) but differs from a study in which an MHR deletion reduced virus production by only 50% (26). This discrepancy could be explained by the finding that overexpression of assembly-defective Gag proteins can result in their release into the medium as particles (M. Tanaka and J. R. Lingappa, unpublished observations), perhaps through production of microvesicles as observed in various disease states (42). Unlike prior studies, our study ensured that microvesicle production was not skewing our data through the use of controls that are known to be assembly defective (reviewed in reference 1) but can be found in the medium when overexpressed, such as the MACA construct (shown in Fig. 2, 4 and 10) and the G2A Gag construct (shown in Fig. 5 to 8).

The current study also provides insights into the relative order in which residues are required for assembly. Our previous studies of assembly-defective HIV-1 Gag mutants (30, 31) demonstrated that mutations within CA helix 9, which forms the interhexameric CA-CTD dimer interface, arrest assembly after Gag has targeted to the membrane but before it has formed a stable membrane-bound complex (class 3 mutants in Fig. 1B [31]); in contrast, mutation of the less conserved K158 residue in the MHR arrests assembly after stabilization of the membrane-targeted Gag oligomer (class 4 mutants in Fig. 1B [31]). Here, we found that MHR deletion or mutation of highly conserved MHR residues arrests assembly at the same step as CA-CTD dimer interface mutants, i.e., after membrane targeting but before stabilization of the Gag oligomer. Thus, mutations in the MHR can result in either class 3 or class 4 defects in the assembly pathway (Fig. 9), arguing that MHR residues are involved in at least two events after membrane targeting, with some residues important for stabilizing the membrane-bound Gag oligomer and another (K158) important for further Gag multimerization at the membrane (Fig. 12A).

Although the function of the MHR is poorly understood, the structure of the MHR is well studied (reviewed in reference 43) and helps to put our data into context. In the crystal structure of the isolated HIV-1 CA-CTD (Fig. 2B), the MHR is preceded by a 3_10_ helix (9). The MHR encompasses a strand-turn-helix structure, in which residues 151 to 155 form the strand, residues 156 to 158 (GPK) form the turn, and residues 161 to 172 form an α-helix (CA helix 8) (8, 9). The strand-turn-helix structure packs against CA helix 9, and this configuration is maintained by hydrogen bonds formed by MHR residues (Fig. 2B), including four of the invariant or highly conserved residues that we studied here (Q155, G156, E159, and R167) (8). Additionally, high-resolution structures of fully assembled immature retroviral capsids confirm that upon completion of immature capsid assembly, CA helix 9 forms the interhexameric CA-CTD dimer interface, while some residues within the MHR either participate in or are in close proximity to intrahexameric CA-CTD interfaces (10, 31, 44) (Fig. 12B). These interfaces are critical for formation of the hexameric subunits that make up the immature HIV-1 lattice. Others have proposed that at some point during assembly the MHR may form a very different “domain-swapped” configuration, in which the MHR, rather than CA helix 9, is the interface between two Gag dimers (45, 46). Such a domain-swapping event would require the MHR to form two different interfaces during assembly and could therefore explain the unique degree of MHR conservation (47); however, to date, no published data exist in support of a domain-swapped configuration for the WT MHR.

Integration of our findings with the results of structural studies described above suggests an explanation for the observed point of arrest in the assembly pathway. Specifically, it is plausible that the point at which these MHR mutants are arrested represents the stage of assembly at which the critical intrahexameric CA-CTD interfaces are formed. If so, that would suggest that the critical intrahexameric CA-CTD interfaces form just after membrane targeting and that formation of these interfaces affects both the stability of the membrane-associated assembly intermediate and its ability to multimerize further at the PM. Additional studies will be required to test this hypothesis.

Finally, analyses of CA-CTD structures reveal that all the invariant and highly conserved MHR residues examined in this study cluster together in the MHR structure (turquoise residues in Fig. 12C). In the CA-CTD crystal structure, the amphipathic CA helix 8 corresponds to nearly two-thirds of the MHR, with uncharged residues along the surface facing CA helix 9 and charged residues along the opposite surface (8, 9). The invariant and highly conserved MHR residues studied here include both charged and uncharged residues; however, together, these occupy only approximately one-third of the surface of the MHR α-helix circumference viewed end-on (turquoise residues in Fig. 12C). Thus, further insights into MHR function could be gained by detailed analysis of residues in the MHR α-helix that were not examined here, including those that are charged versus uncharged (dark blue versus red residues, respectively, in Fig. 12C).
